# Safety of temporary ileostomy via specimen extraction site in rectal cancer patients who underwent laparoscopic low anterior resection

**DOI:** 10.1038/s41598-019-38790-6

**Published:** 2019-02-19

**Authors:** Kil-yong Lee, Ji Won Park, Ki-young Lee, Sangsik Cho, Yoon-Hye Kwon, Min Jung Kim, Seung-Bum Ryoo, Seung-Yong Jeong, Kyu Joo Park

**Affiliations:** 1 Department of Surgery, Seoul National University College of Medicine, Seoul, Korea; 2 Cancer Research Institute, Seoul National University College of Medicine, Seoul, Korea

## Abstract

If anastomotic site leakage is expected after laparoscopic low anterior resection (LAR), de-functioning ileostomy is required. However, there is controversy about the consequence of stoma formation via the specimen extraction site (SES). Therefore, we aimed to investigate stoma-related complication according to stoma formation via the SES. We enrolled rectal cancer patients who underwent laparoscopic LAR with temporary ileostomy between January 2013 and December 2017. Patients were divided into two groups: stoma through the SES (SES) and stoma through a new site (NS). The difference in the incidence of stoma-related complications was analysed. In total, 198 patients underwent laparoscopic LAR (SES = 141 patients, NS = 57 patients). The SES group had a shorter operation time (204.7 ± 74.4 min vs 229.5 ± 90.5 min, p = 0.049) and was associated with fewer cases of wound infection (0% vs 7%, p = 0.006) than the NS group. There was no statistically significant difference between the SES group and NS group in all-stoma complications (22.7% vs 12.3%, p = 0.095). The incidence of parastomal hernia also was not significantly different (11.3% vs 5.3%, p = 0.286). Stoma via the SES is feasible after laparoscopic LAR with temporary ileostomy, although stoma-related complication rate was higher, without a significant difference. It can shorten the operation time and reduce wound infection rate.

## Introduction

Since the introduction of abdominal surgery using the laparoscopic approach as minimally invasive surgery, the incidence of postoperative pain and adhesion and the length of hospital stay have decreased compared to using open surgery. Laparoscopic operations have become increasingly popular because of their cosmetic effects^1^. Since researchers reported no difference in oncologic outcomes between open surgery and laparoscopic surgery in low anterior resection (LAR) for patients with rectal cancer^2–4^, laparoscopic surgery has gradually replaced open surgery.

When performing laparoscopic LAR, de-functioning stoma is considered to prevent pelvic sepsis for patients who have low anastomosis levels, who underwent neoadjuvant concurrent chemoradiotherapy, or who are at high risk of anastomotic site leakage due to poor vascularity^5–7^. Additional incision is needed to perform specimen extraction following a laparoscopic operation, and there is a controversy regarding where de-functioning stoma should be performed with respect to the incision. Li *et al*. claimed that forming a stoma at the SES can increase the risk of stoma-related complications and that it is safe to perform ileostomy at a different location^8^. However, research on complication rates when a stoma is formed at the SES in patients with rectal cancer is lacking. Therefore, this study aimed to compare the differences in complication rates, such as parastomal hernia and stoma prolapse, based on whether a temporary ileostomy was created at the site of specimen extraction.

## Materials and Methods

This retrospective study was approved by the Seoul National University Hospital Institutional Review Board (SNUH IRB). The study protocol was performed in accordance with the guidelines and regulation of the SNUH IRB. Written informed consent was waived after being reviewed by the SNUH IRB.

### Patients and operative procedure

Among patients with rectal cancer who underwent LAR using laparoscopy between January 2013 and December 2017, those who underwent temporary de-functioning ileostomy were included. The included patients were divided according to the site of stoma formation: a stoma at a new site (NS group) and a stoma at the specimen extraction site (SES group). Follow-up was also conducted. During the surgery, a multiport laparoscopic technique (five holes incision) was used. Following the ligation of the inferior mesenteric artery and inferior mesenteric vein, colon mobilisation and total mesorectal excision were performed. Then, an extended incision was created at a subumbilical, left lower quadrant (LLQ) or right lower quadrant (RLQ) port site on the specimen, and a wound protector was used to retract it. The average length of the extended incision was 6 cm. Thereafter, extracorporeal extraction was performed followed by coloanal anastomosis using the double stapling method or the hand-sewn method. In the SES group, fascia closure was performed after leaving a trephine of sufficient width for two fingers at the site of specimen extraction, and an ileostomy was formed at the same site. In the NS group, additional incision was made at a port site different from the SES, and a stoma was formed.

### Follow-up

The incidence of immediate complications such as postoperative ileus, urinary retention, wound, and cardiopulmonary complications that occurred during the hospitalisation period was investigated. Patients were discharged if they adhered to a normal bland diet or normal regular diet and showed no unusual symptoms. The patients visited the hospital at 2 weeks after surgery for stoma inspection. They visited an outpatient clinic for cancer surveillance and had additional checkups on stoma-related complications. Patients who required additional anticancer treatment underwent ileostomy repair after the treatment. Patients who did not undergo anticancer treatment underwent ileostomy repair about 3 months later.

### Variables

Clinical characteristics, operative and postoperative outcomes, pathologic findings, and stoma-related complications were retrospectively investigated using medical records. Time to first flatus was investigated by identifying whether gas was escaping through the ileostomy after surgery. Postoperative pain was measured by the nurse in charge using the numerical rating scale (NRS). The incidence of parastomal hernia, which is one of the stoma-related complications, was investigated using computed tomography (CT) for patients who underwent CT for cancer surveillance.

### Statistical analysis

A chi-square test or Fisher exact test was used to compare categorical variables, and a t-test was used to compare continuous variables between the two groups. For univariable analysis on the factors that affected the incidence of stoma site complications, the chi-square, Fisher exact, and Mann-Whitney tests were used. For factors with p < 0.2, a logistic regression test was used for the multivariable analysis. Statistical tests were performed using the Statistical Package for the Social Sciences version 22.0 for Windows (IBM Corp, Armonk, NY, USA) and p < 0.05 was considered statistically significant.

## Results

A total of 198 patients underwent laparoscopic LAR with temporary ileostomy. Of these patients, 141 were in the SES group where the stoma site and site of specimen extraction were the same, and 57 were in the NS group where the site of specimen extraction differed from the stoma site. The baseline characteristics of the two groups are shown in Table 1.

**Table 1:** Baseline characteristics.

	Stoma at a new site (NS) (n = 57)	Stoma at the specimen extraction site (SES) (n = 141)	p-value
Age	63.7 ± 10.9	62.4 ± 11.4	0.482
Sex			0.888
Male	37 (64.9%)	93 (66.0%)	
Female	20 (35.1%)	48 (34.0%)	
BMI (kg/m^2^)	23.9 ± 1.0	23.7 ± 0.8	0.671
ASA class			0.071
1	9 (15.8%)	44 (31.2%)	
2	45 (78.9%)	89 (63.1%)	
3	3 (5.3%)	8 (5.7%)	
Diabetes	16 (28.1%)	27 (19.1%)	0.168
HTN	22 (38.6%)	54 (38.3%)	1
COPD	0 (0%)	3 (2.1%)	0.558
Smoking history			0.272
Past smoker	5 (8.8%)	15 (10.6%)	
Current smoker	8 (14.0%)	28 (19.9%)	
Preoperative chemoradiotherapy	29 (50.9%)	66 (46.8%)	0.604

ASA, American Society of Anesthesiologists; BMI, body mass index; COPD, chronic obstructive pulmonary disease; HTN, hypertension.

Moreover, 134 patients in the SES group (95%) had specimen extraction at the LLQ. In the NS group, 48 patients (84.2%) underwent specimen extraction in the periumbilical area, excluding nine patients who underwent coloanal anastomosis following transanal specimen extraction. Regarding stoma formation, an ileostomy was formed at the RLQ in 56.1% of patients in the NS group. Operation time was significantly shorter in the SES group than in the NS group (204.7 vs 229.5 min, p = 0.049) (Table 2).

**Table 2:** Operative findings.

	Stoma at a new site (NS) (n = 57)	Stoma at the specimen extraction site (SES) (n = 141)	p-value
Specimen extraction site			0.004
Periumbilical	48 (84.2%)	3 (2.1%)	
LLQ	0 (0%)	134 (95.0%)	
RLQ	0 (0%)	4 (2.8%)	
Anus	9 (15.8%)	0 (0%)	
Ileostomy site			0.000
LLQ	24 (42.1%)	134 (95.0%)	
RLQ	23 (56.1%)	7 (5.0%)	
RUQ	1 (1.8%)	0 (0%)	
Operation time (min)	229.5 ± 90.5	204.7 ± 74.4	0.049
Estimated blood loss (cc)	216.6 ± 331.0	151.4 ± 155.1	0.159

LLQ, left lower quadrant; RLQ, right lower quadrant; RUQ, right upper quadrant.

There was no significant difference in the postoperative pain score, time to first flatus, and length of stay between the two groups. Most patients did not show postoperative complications. Four patients in the NS group showed wound infection at a statistically significant rate (p = 0.006) (Table 3).

**Table 3:** Postoperative outcomes.

	Stoma at a new site (NS) (n = 57)	Stoma at the specimen extraction site (SES) (n = 141)	p-value
Postoperative pain score			
First day	5.0 ± 0.9	5.1 ± 1.2	0.595
Second day	4.3 ± 0.8	4.1 ± 0.9	0.184
Third day	3.6 ± 0.6	3.6 ± 0.8	0.933
Time to first flatus	2.1 ± 1.0	1.9 ± 0.8	0.482
Length of stay	7.0 ± 3.7	7.1 ± 2.7	0.813
Postoperative complication			
Ileus	9 (15.8%)	15 (10.6%)	0.315
Cardiopulmonary	0 (0%)	1 (0.7%)	1
Wound infection	4 (7%)	0 (0%)	0.006
Urinary retention	3 (5.3%)	10(7.1%)	0.761
Postoperative transfusion	4 (7.0%)	2 (1.4%)	0.058
Time to ileostomy reversal (day)	180.7 ± 81.7	170.4 ± 74.3	0.399

For pathologic findings, there was no significant difference in the tumour size and the incidence of harvested lymph node, metastatic lymph node, and resection margin between the two groups. There was also no significant difference in the pathologic stage and incidence of lymphatic, venous, and perineural invasion (Table 4).

**Table 4:** Pathologic outcomes.

	Stoma at a new site (NS) (n = 57)	Stoma at specimen the extraction site (SES) (n = 141)	p-value
Tumour size (cm)	3.2 ± 1.9	3.0 ± 2.0	0.489
Metastatic lymph node	1.2 ± 2.3	0.7 ± 2.0	0.143
Harvested lymph node	19.7 ± 8.1	19.4 ± 8.6	0.867
Resection margin			
Proximal (cm)	14.5 ± 9.8	11.9 ± 5.3	0.069
Distal (cm)	2.5 ± 3.6	1.8 ± 2.3	0.202
Radial (mm)	8.4 ± 5.5	10.7 ± 20.7	0.44
Stage (pTNM)			0.189
0	5 (8.8%)	17 (12.1%)	
1	15 (26.3%)	46 (32.6%)	
2	15 (26.3%)	38 (27.0%)	
3	19 (33.3%)	32 (22.7%)	
4	3 (5.3%)	8 (5.7%)	
Lymphatic invasion	9 (15.8%)	26 (18.4%)	0.658
Venous invasion	7 (12.3%)	16 (11.4%)	0.866
Perineural invasion	18 (31.6%)	42 (29.8%)	0.804

Although stoma-related complications occurred more frequently in the SES group, there was no significant difference in the incidence of these complications between the two groups (12.3% vs 22.7%, p = 0.095) (Table 5). Parastomal hernia, the most common complication, occurred more frequently in the SES group (n = 16, 11.3%) than in the NS group (n = 3, 5.3%), but did not show a significant difference (p = 0.286). During follow-up, 153 patients (77.3%) underwent follow-up CT, and 19 patients showed parastomal hernia on CT. Regarding re-operation due to stoma problems, two patients (1.4%) had stoma formation at SES, and one patient had stoma formation at a NS (1.8%). The incidence of re-operation due to stoma problems was not statistically significant (p = 1.000).

**Table 5:** Stoma-related complication.

	Stoma at a new site (NS) (n = 57)	Stoma at the specimen extraction site (SES) (n = 141)	p-value
Parastomal hernia	3 (5.3%)	16 (11.3%)	0.286
Stoma prolapse	2 (3.5%)	7 (5.0%)	1.000
Skin inflammation	2 (3.5%)	8 (5.7%)	0.727
Retraction	0 (0%)	1 (0.7%)	1.000
Bleeding	1 (1.8%)	0 (0%)	0.288
All-stoma complication	7 (12.3%)	32 (22.7%)	0.095

Ileostomy reversal was performed in 188 patients (94%). The mean duration from stoma formation to reversal in SES and NS was 180.7 and 170.4 days, respectively (Table 3). One patient in the NS group did not undergo reversal of ileostomy because the patients who had stage IV required continuous chemotherapy. Nine patients in the SES group did not undergo ileostomy reversal because of the following: died while planning reversal (two patients), on-going chemotherapy because of tumour stage (four patients), anastomosis site problem (two patients), and patient refusal (one patient).

In the univariate analysis, no factors could be identified that significantly affected the incidence of stoma-related complications. However, when a multivariate analysis was performed with variables with p < 0.2 including age (p = 0.102), sex (p = 0.083), SES stoma (p = 0.095), hypertension (HTN) (p = 0.069), and smoking status (p = 0.105), whether a stoma was formed at the SES did not significantly affect the incidence of stoma-related complications (odds ratio (OR) = 2.184, 95% confidential index (CI) 0.0890–5.359, p = 0.088).

In the multivariate analysis performed to identify the factors that affect the incidence of parastomal hernia, age (p = 0.044), BMI (p = 0.010), and HTN (0 = 020) were identified as significant factors. However, in a logistic regression test using factors including SES stoma, BMI was identified as a significant factor (OR 1.18, 95% CI 1.010–1.387, p = 0.037), but age (OR 1.03, 95% CI 0.980–1.085, p = 0.240), SES stoma (OR 2.69, 95% CI 0.715–10.087, p = 0.144), and HTN (OR 2.0, 95% CI 0.652–6.134, p = 0.226) were not.

## Discussion

In this study, we demonstrated that stoma formation at the SES in laparoscopic LAR and temporary de-functioning ileostomy had more stoma-related complication than the NS group even though there was no significant difference. However, given the shorter operation time in the SES group compared to the NS group, better outcomes may be obtained by forming a stoma at the SES.

Moreover, forming a stoma at SES reduces the overall wound length, thereby reducing the time required for wound closure, and reduces postoperative pain^9^. However, stoma formation at the SES reduced operation time, but not postoperative pain in this study. This may be because the NRS lack objectivity in measuring postoperative pain as a self-report system.

To the best of our knowledge, there were three studies on ileostomy formation at the SES. Karakayali *et al*. reported that stoma formation at the SES could minimise incisions, which improved cosmesis but may increase the incidence of parastomal hernia (19%) in 21 patients with de-functioning ileostomy at the SES and 25 patients with ileostomy at a new incision^10^. Wang *et al*. reported that ileostomy formation via the SES was a feasible method because that group had a shorter operation time, less estimated blood loss, and less wound complication rate than the control group^9^. However, Li *et al*. reported that ileostomy formation at the SES should be performed with caution because of increased incidence of stoma site complications^8^. In this study, temporary ileostomy formation at the SES was associated with a shorter operation time and less wound complication rate than the control group, which is similar to that reported by Karakayali *et al*. and Wang *et al*. Although the incidence of overall stoma site complication rate in SES was higher than that in NS, which is similar to that reported by Li *et al*., there was no significant difference between the two groups.

We created a temporary ileostomy by extending an incision at the LLQ port site in most patients who had a stoma formed at the SES. Since there was no significant difference in the postoperative outcome between when an ileostomy site was formed on the left side or the right side and since extracting the specimen from the right site requires more dissection than extracting it from the left site for colon mobilisation^11^, we performed specimen extraction and stoma formation on the left side for most patients. Wang *et al*.^9^ and Karakayali *et al*.^10^ made the stoma in RLQ and Li *et al*.^8^ did not specify the site. In this respect, we can distinguish the stoma formed. We confirmed the safety of the procedure through LLQ.

The most common complication that occurs at the SES is parastomal hernia^8^, and this was proven in our study. This study included patients with cancer, and for those with advanced cancer, stoma reversal was performed after the patients completed chemotherapy, and cancer surveillance CT was performed during this process. Although the patients did not show symptoms of parastomal hernia, cases in which parastomal hernia was incidentally detected on CT were included, and this may have led to the higher incidence of hernia than that reported by Wang *et al*.^9^. This study provides data regarding the incidence of parastomal hernia that occurred after stoma formation at the SES that are more objective than those provided by Wang *et al*.^9^, Karakayali *et al*.^10^ and Li *et al*.^8^ whose studies were based on symptoms.

Although SES stoma formation, transfusion, and BMI have been reported to affect the incidence of stoma-related complications^8^, stoma formation at the SES did not affect the incidence of stoma-related complications in this study. In the multivariable analysis on the factors that affected the incidence of parastomal hernia, BMI was identified as a significant factor, and this was consistent with previous reports^12,13^.

This study has some limitations. First, this study may contain bias as data were retrospectively collected. Second, the possibility of type II errors due to the relatively small number of subjects in the NS group, as a reason for the lack of difference in the incidence of stoma-related complications between the two groups, cannot be eliminated. However, considering that the patients included in this study had a temporary ileostomy and the incidence of severe complications requiring re-operation was low, the difference in the incidence of stoma-related problems can be overcome.

In conclusion, when performing laparoscopic LAR with temporary de-functioning ileostomy, it is feasible to form a stoma at the SES. It can shorten the operation time and reduce wound infection rate; although stoma-related complication rate in the SES was higher than that in an NS, there was no significant difference. In particular, this technique is worth considering in minimally invasive procedures, as a better cosmetic effect can be expected by reducing the size of the wound. In the future, the safety of temporary ileostomy via the SES needs to be further strengthened through a randomised controlled study.

## References

[1] Sabiston, D. C. & Townsend, C. M. Sabiston textbook of surgery: the biological basis of modern surgical practice. 19th edn, (Elsevier Saunders, 2012).

[2] Fleshman J (2015). Effect of laparoscopic-assisted resection vs open resection of stage II or III rectal cancer on pathologic outcomes: the ACOSOG Z6051 Randomized Clinical Trial. JAMA.

[3] Green BL (2013). Long-term follow-up of the Medical Research Council CLASICC trial of conventional versus laparoscopically assisted resection in colorectal cancer. Br J Surg.

[4] Kang SB (2010). Open versus laparoscopic surgery for mid or low rectal cancer after neoadjuvant chemoradiotherapy (COREAN trial): short-term outcomes of an open-label randomised controlled trial. Lancet Oncol.

[5] Gu WL, Wu SW (2015). Meta-analysis of defunctioning stoma in low anterior resection with total mesorectal excision for rectal cancer: evidence based on thirteen studies. World J Surg Oncol.

[6] Matthiessen P, Hallbook O, Rutegard J, Simert G, Sjodahl R (2007). Defunctioning stoma reduces symptomatic anastomotic leakage after low anterior resection of the rectum for cancer: a randomized multicenter trial. Ann Surg.

[7] Tan WS, Tang CL, Shi L, Eu KW (2009). Meta-analysis of defunctioning stomas in low anterior resection for rectal cancer. Br J Surg.

[8] Li WL (2017). Does stoma site specimen extraction increase postoperative ileostomy complication rates?. Surg Endosc.

[9] Wang P, Liang JW, Zhou HT, Wang Z, Zhou ZX (2018). Surgical specimen extraction via a prophylactic ileostomy procedure: a minimally invasive technique for laparoscopic rectal cancer surgery. World J Gastroenterol.

[10] Karakayali FY (2015). Specimen extraction from the defunctioning ileostomy site or Pfannenstiel incision during total laparoscopic low anterior resection for rectal cancer. J Laparoendosc Adv Surg Tech A.

[11] Yoo SB (2013). Left-sided ileostomy at specimen extraction site in laparoscopic-assisted low anterior resection for rectal cancer. J Laparoendosc Adv S.

[12] Duchesne, J. C., Wang, Y. Z., Weintraub, S. L., Boyle, M. & Hunt, J. P. Stoma complications: a multivariate analysis. *Am Surg* **68**, 961–966; discussion 966 (2002). 12455788

[13] Nastro P (2010). Complications of intestinal stomas. Br J Surg.

